# Energy Optimization of Wireless Sensor Embedded Cloud Computing Data Monitoring System in 6G Environment

**DOI:** 10.3390/s23021013

**Published:** 2023-01-16

**Authors:** Huaiyuan Yang, Hua Zhou, Zhenyu Liu, Xiaofan Deng

**Affiliations:** 1 College of Big Data and Intelligent Engineering, Southwest Forestry University, Kunming 650224, China; 2 School of Software and Internet of Things Engineering, Jiangxi University of Finance and Economics, Nanchang 330000, China

**Keywords:** ZigBee technology, energy optimization, wireless sensor network, embedded cloud computing, monitoring system, 6G environment

## Abstract

With the construction and development of modern and smart cities, people’s lives are becoming more intelligent and diversified. Surveillance systems increasingly play an active role in target tracking, vehicle identification, traffic management, etc. In the 6G network environment, facing the massive and large-scale data information in the monitoring system, it is difficult for the ordinary processing platform to meet this computing demand. This paper provides a data governance solution based on a 6G environment. The shortcomings of critical technologies in wireless sensor networks are addressed through ZigBee energy optimization to address the shortage of energy supply and high energy consumption in the practical application of wireless sensor networks. At the same time, this improved routing algorithm is combined with embedded cloud computing to optimize the monitoring system and achieve efficient data processing. The ZigBee-optimized wireless sensor network consumes less energy in practice and also increases the service life of the network, as proven by research and experiments. This optimized data monitoring system ensures data security and reliability.

## 1. Introduction

**Figure 1:**
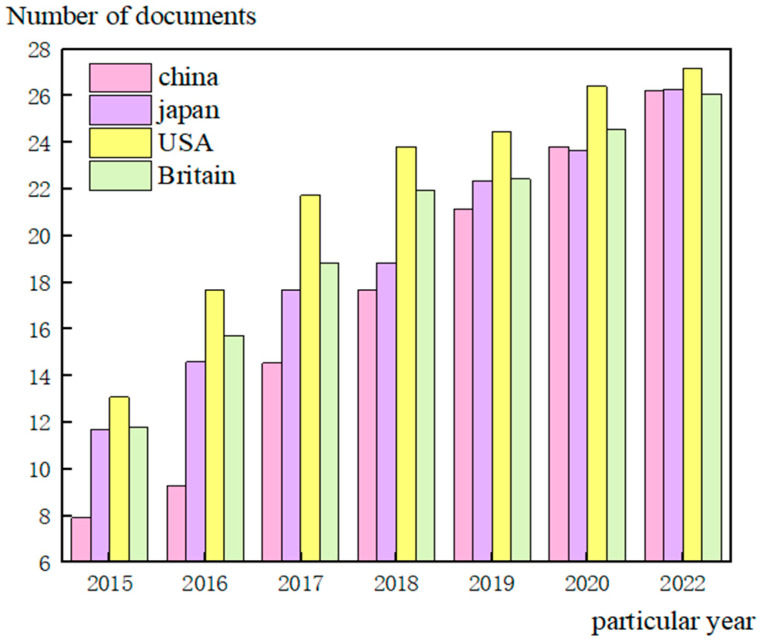
Case statistics of monitoring research using wireless sensor networks in different countries.

With the acceleration of urban modernization, people pay more attention to improving safety control awareness. The monitoring system has become essential to ensure people’s daily travel and traffic safety [1]. The transformation from video surveillance to digital data surveillance has also added multiple changes to this field. More and more monitoring systems are being applied to military, medical, target detection, traffic, environmental monitoring, and other industries [2]. Massive data processing has brought computing obstacles to the traditional monitoring system platform, which has a challenge coping with the processing needs of large amounts of data [3]. Therefore, wireless sensor networks and embedded cloud computing, as a way of distributed real-time computing, have gradually become effective solutions for processing and monitoring data [4]. The embedded cloud computing platform based on big data has a high-efficiency requirement when processing data input and output. Using a distributed structure to process the collected data information synchronously, this intelligent multi-processing task mode also brings a significant energy consumption. The use of wireless sensor networks as the infrastructure of the monitoring system has also been a hot topic of research in various countries in recent years. We provide statistics on the cases of different countries using wireless sensor networks for monitoring research, as shown in Figure 1:

It can be seen from Figure 1 that there are many types of research in wireless sensor networks in the United States, the United Kingdom, and other Western countries. This situation depends on their computer technology development and communication technology development. China and Japan, two Asian countries, have also reflected the change in research quantity according to their development [5]. The advantages of a wireless sensor network lie in its low price, convenient deployment of nodes, and rapid data flow in a short distance. However, its most significant disadvantages are its limited energy, high maintenance cost, and poor storage and computing capacity.

With the maturity of the 5G standard and the gradual expansion of commercial applications, 6G has quickly entered the industry’s vision. Many institutions and organizations have released white papers on 6G technology, pointing out the direction for future 6G technology indicators and applications. However, 5G is still in the primary stage of commercial application. There is still great uncertainty as to whether 5G supports various vertical applications. Relevant technologies are not yet mature, and business models still need to be explored. Any generation of systems has revolutionary changes in wireless access technology or network architecture. The development of mobile communication has the characteristics of continuity. Therefore, while discussing 6G, we should also think about how to effectively continue the development of mobile communications based on 5G and, at the same time, continue to absorb advanced technologies to continuously improve the technical content of mobile networks and reduce the use cost. Under such limited conditions, the large-scale data transmitted by embedded and cloud computing platforms quickly cause data congestion in wireless sensor networks, increasing the energy consumption of transmission nodes and causing network paralysis at any time [6].

Therefore, we found from the literature survey that ZigBee energy optimization technology can solve the above problems well. As a new communication technology with low power consumption, high efficiency, and a faster speed, this technology reduces network delay to a specific range by its advantages. Additionally, it improves the data storage capacity limit in wireless sensor networks [7]. With the development of the Internet of Things and communication sensor technology, ZigBee technology has also optimized the energy defect problem in wireless sensor networks. It has been applied in many fields, such as intelligent medical treatment, industrial management, traffic control, etc. [8]. Its characteristic is that it can work and stand by in the form of low consumption to ensure the system can run for a longer time. This technology is relatively simple in terms of protocol, reducing communication control requirements and saving the use cost. Finally, it has a meager delay rate, communication speed is breakneck, and it can also meet the fast transition between work and sleep [9]. ZigBee technology guarantees data storage capacity based on various network structures and optimizes and complements wireless sensor networks’ energy supply and storage defects [10]. Therefore, if the ZigBee energy-optimized sensor network is added to the embedded cloud computing platform, it can improve the data monitoring system’s efficiency in transmitting information and increase the storage capacity and service life of the system.

The research innovation contribution lies in solving the problem of insufficient energy supply and high power consumption in the practical application of wireless sensor networks. ZigBee energy optimization is used to solve the defects of critical technologies in wireless sensor networks by using the embedded cloud computing platform to process data in a distributed manner, and forming a multi-point computing structure based on real-time processing virtualization and integration. The redundancy in network data operation is reduced, and the problem of excessive energy consumption in wireless sensor network applications is reduced. Through a simple analysis of ZigBee technology, a clustering algorithm is proposed to optimize the routing protocol with high energy consumption. In the optimization process, the AODVjr algorithm is added as the comparison result of energy consumption. Adding dynamic monitoring data to the multi-task joint processing process improves the cloud storage capacity of the monitoring system and shortens the response time for processing monitoring data. In actual use, it consumes less energy and improves the service life of the network. This optimized data monitoring system ensures the security and reliability of data.

Section 1 describes the research background of using wireless sensor networks as the monitoring system’s infrastructure. The energy deficiency in existing wireless sensor networks is analyzed. Section 2 describes the development status of ZigBee technology in various countries. It has been the research achievement of many scholars to improve the operation efficiency and availability of monitoring networks. Section 3 studies the embedded cloud computing data monitoring system based on the ZigBee energy-optimized wireless sensor network routing algorithm. At the same time, the simulation of a ZigBee-optimized wireless sensor network in an embedded cloud computing data monitoring system is carried out. Section 4 analyzes the simulation results of the wireless sensor network routing algorithm based on ZigBee energy optimization. With the increase in the system’s response time in data processing, the interference effect occurs ahead of time for the ordinary wireless network and Bluetooth transmission. The wireless sensor network optimized by ZigBee technology has longer anti-interference time and more apparent advantages than the other two. Section 5 summarizes the full text. Using ZigBee energy to optimize wireless sensor networks in embedded cloud computing data monitoring systems can improve system operating efficiency. It not only reduces the running loss of the system but also ensures the security of the data and realizes a wide range of monitoring coverage.

## 2. Related Work

In the future, the 6G spectrum will be extended to terahertz. In terms of coverage, 6G will use satellite communication, UAV communication, new airport technology, etc., to realize the seamless coverage of the communication network in the air, space, earth, and sea and the wide-area intelligent connection at the global business level of people, machines, and things. Therefore, the 6G network needs to be deeply integrated with computing to complete the integration and access of massive heterogeneous networks and allocate and cooperate full-time and frequently with airspace resources. In addition, it can meet the requirements of an intelligent, ubiquitous, and secure connection for the seamless coverage communication of 6G space, space, sea, and human–machine and object. In the Internet of Things and communication era, digital life affects people’s behavior changes [11]. Everyone has higher and higher requirements for wireless sensing and communication. As an indispensable part of social security, the monitoring system needs to be improved and optimized by using new means [12].

FAJIT is mainly committed to solving the problem of parent node selection in heterogeneous networks to different aggregate types of data packets to improve energy efficiency. Bhushan et al. compared the results of FAJIT with the distributed algorithm for integrated tree construction and data aggregation (DICA) on various parameters: average scheduling length, energy consumption data interval, the total number of transmission time slots, control overhead, and energy consumption in the control phase. The results show that the proposed algorithm has better energy efficiency [13]. The emergence of wireless networks and other technologies has led to the rapid development of communication technology [14]. The generation of various data has also brought trouble to the operation of the monitoring system platform. Therefore, how to improve data processing and improve the operation efficiency and availability of the monitoring network has been the focus of many scholars. As a comprehensive international discipline, the wireless sensor network contains sensor technology, embedded processing technology, cloud computing technology, big data technology, communication technology, etc. It has been widely used in all walks of life [15,16]. This wireless sensor network is composed of microsensors when nodes are deployed, and finally, a monitoring area is formed by network nodes. The monitoring network formed by many monitoring systems also uses a wireless communication mode to form a multi-structure, adaptive distribution area. It completes target detection through real-time data acquisition and processing [17]. A large amount of information generated by these monitoring objects needs to be added to the terminal platform for analysis and processing and finally sent to the monitoring system center to complete the corresponding tasks. However, this single wireless sensor network often needs more energy supply and a slow processing speed, which makes it easy to make mistakes in the face of a large number of dynamic data collected by the monitoring system. Fuzzy multi-cluster routing based on a constant threshold (FMCR-CT) is a solution that can save more energy in wireless sensor networks. Most of the algorithms introduced so far are based on each round of clustering and single-hop data transmission to the base station. Each round of clustering increases the number of control messages and the possibility of conflicts. FMCR-CT is innovative in many areas, including avoiding clustering in each round, introducing fixed thresholds, using different algorithms for clustering, and using multi-hop routing by considering appropriate intermediate nodes to send data from each cluster to the base station. The primary purpose of Mazinani et al.’s research was to propose a method to improve the lifetime of wireless sensor networks by reducing cluster head selection and messages transmitted in each round [18].

**Figure 2:**
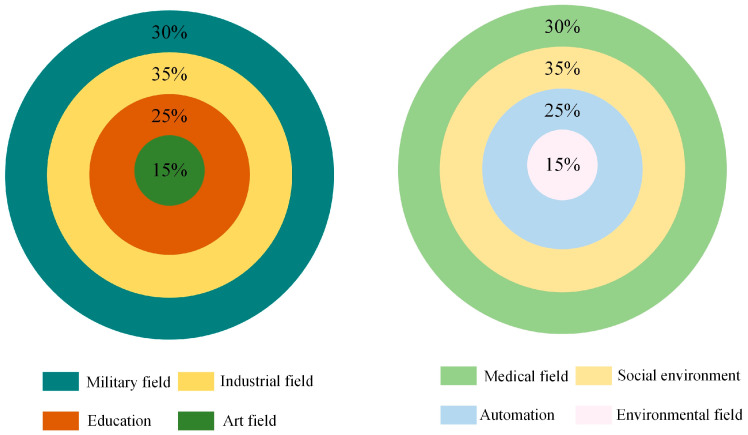
Frequency distribution of application fields of this technology in various countries.

ZigBee technology is a wireless technology with high efficiency, low latency, low cost, and long duration. It conforms to the specification of network control protocol and is applied to sensor networks and remote control in the form of the complete structure. This new wireless communication technology uses a free frequency band for signal transmission, which is simple and consumes less energy [19]. The wireless sensor network (WSN) is one of the most active research fields. They have many applications, such as military, medical, environmental monitoring and industrial monitoring. The energy of sensor nodes is limited. In many cases, the node is deployed in an unreachable area, so the battery of the sensor node cannot be charged or replaced. Therefore, technologies must be adopted to save energy by reducing the energy consumption of nodes. Chowdhury and Hossain discussed different energy-saving schemes studied by different research groups in wireless sensor networks to reduce the energy consumption of nodes and thus improve the life of the entire network. Energy-saving protocols such as duty cycle, energy-saving routing, energy-saving medium access control (MAC), data aggregation, cross-layer design, and error control code (ECC) have been discussed. The duty cycle method uses the sleep/wake method to reduce the activity time of nodes and save their energy [20]. We investigated the frequency distribution of the application fields of this technology in various countries, as shown in Figure 2.

It can be seen from Figure 2 that ZigBee technology, as a high-performance wireless communication technology, is mainly used in military and industrial fields in Western countries. It is widely used in medicine, social environment, automation, and other fields in Asian countries. We analyzed the effect of using ZigBee technology in the United States in detail and found that they had carried out fusion applications since the smart sensor stage. At first, this kind of low-energy consumption wireless sensor was used in the military field [21]. The US Defense Department has provided financial support for wireless sensor networks and is determined to take sensor research as the long-term planning goal. Based on ZigBee technology’s high efficiency and low latency, the strategic communication network is optimized, which helps the information transmission between the front-line forces and the logistics forces [22]. Although this technology has developed rapidly in Western countries, Japan has also effectively combined ZigBee technology with intelligent society. They found that when intelligent transportation and intelligent furniture are integrated into the sensor network, they can improve the interaction between people and objects. At the same time, in the design of power-saving infrastructure, this controllable and low energy consumption technology is combined with the street lamp system to obtain a good optimization effect [23].

In some areas with desired and sufficient coverage, the operation schedule of the node is set to activate some sensors to ensure necessary coverage and disable other sensors. This has dramatically reduced the energy demand. A mobile node improves coverage when a cell is deprived of proper or sufficient coverage. The method proposed by Nilsaz and Barati has more advantages than its comparative technology, especially in the area ratio of a network domain, energy consumption, and network life [24]. Karimi et al. studied a heterogeneous two-layer wireless sensor network in which N heterogeneous access points (APs) collect sensing data from densely distributed sensors. The simulation results show that the proposed algorithm is better than the existing clustering methods, such as the node layout in the minimum energy routing and the improved node layout [25]. Guleria and Verma discussed layered energy-efficient routing protocols based on classical and swarm intelligence methods. We can summarize these two routing protocols according to energy efficiency, data aggregation, location awareness, QoS, scalability, load balancing, fault tolerance, query-based, and multipath. The layered routing protocol was studied and analyzed to improve energy efficiency and network life [26]. The wireless sensor network (WSN) is a group of small power-limited nodes that sense data and transmit them to the base station (BS). Although the energy introduced in the setup phase and the energy fairness constraint of the dynamic routing topology is violated, they achieve high network performance in terms of coverage and connectivity. Mohamed et al. classified the applications of wireless sensor networks according to different aspects to show the main protocol design problems. Therefore, the energy efficiency of current active routing protocols has been studied from different perspectives. The most energy-efficient routing protocols, inhomogeneous active networks, have been studied and compared. The results show that energy cost and routing are the most compelling aspects of network life and efficiency [27].

Therefore, in our design of the data monitoring system, adding the above technologies is also a critical scheme to increase the system operation time and improve the data processing speed and stability.

## 3. Analysis of Embedded Cloud Computing Data Monitoring System Based on Wireless Sensor Network

### 3.1. Simulation of Wireless Network Algorithm Based on ZigBee Energy Optimization

**Figure 3:**
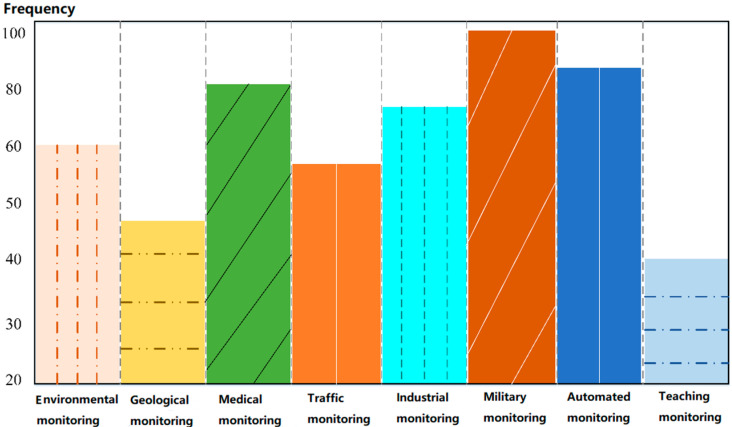
The usage frequency of wireless sensor networks in various fields.

In 6G applications, the impact of the access side will be more and more far-reaching, and the requirements for the rate and delay of service applications will become greater and greater. The role of embedded cloud computing in wireless sensor network routing algorithms will also be more prominent [28]. The wireless sensor network is a communication network composed of an unfixed network topology. Daily, many monitoring areas are deployed with monitoring nodes formed by microsensor networks. These sensor nodes use frequency hopping to transmit signals and data and rely on cooperation between sensor nodes to complete data acquisition, sensing, processing, and other tasks [29]. Finally, network coverage sends the collected data and signals to the monitoring center in a dynamic transmission mode. In practical applications, wireless sensor networks do not need stable and specialized network support and can work quickly in any environment [30]. According to our research in the literature, the frequency of use of wireless sensor networks in various fields is shown in Figure 3.

**Figure 4:**
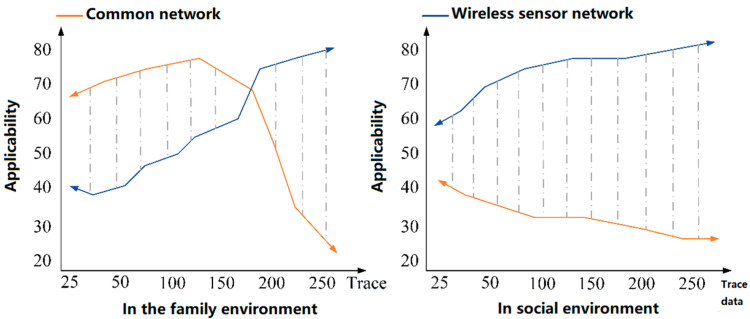
Comparison of applicability between family and social networks.

It can be seen from Figure 3 that wireless sensor networks are well used in military monitoring, medical health monitoring, and fire monitoring. With the construction of modern cities, wireless sensor networks are also used in the research of traffic management and road monitoring [31]. This new data and information processing mode has become the key research object in the monitoring field. The gap between wireless sensor networks and traditional networks is obvious. In the same data calculation process, if you want to ensure the quality of data transmission, select wireless sensor networks. Therefore, in the face of dynamic information in a complex network environment, wireless sensor networks have more advantages than ordinary networks [32]. We compare the applicability of the two networks from the user traces of family and society, as shown in Figure 4.

It can be seen from Figure 4 that the use frequency of ordinary networks in the home environment is high at the beginning. However, it changes with the optimization of wireless sensor networks. In the social environment, users prefer wireless sensor networks. This network structure is small and efficiently uses energy and bandwidth for computing data. However, wireless sensor networks still have their shortcomings, such as the storage capacity, and a large amount of dynamic data computing is prone to failure [33]. In the monitoring system, the service life of the wireless sensor network is affected by energy consumption, which cannot ensure the long-term stable operation of the system. At the same time, the energy supplement of wireless sensor networks is troublesome, and the maintenance cost is also high. Therefore, we add ZigBee energy optimization technology to our research as the best way to improve wireless sensor networks [34]. ZigBee technology is a wireless communication technology with low speed, high efficiency, and short distance, which applies to data transmission, information transmission, path tracking, and detection. Its advantages lie in its lower energy consumption, lower cost of design and deployment, and longer transmission distance than ordinary wireless sensor networks. At the same time, the system has significant advantages in terms of activation speed and sleep delay [35]. The network structure can be distributed into a tree type, star type, etc. When processing a large amount of data and data in the same direction, the wireless sensor network under ZigBee technology can effectively avoid the collision time of multiple data. It can resend the data after detecting the data transmission problem [36]. Therefore, ZigBee technology has a strong application capability, and the system also guarantees the security of data transmission. We compare the performance of ZigBee technology with Bluetooth transmission, wireless network transmission, and other methods, as shown in Table 1.

**Figure 5:**
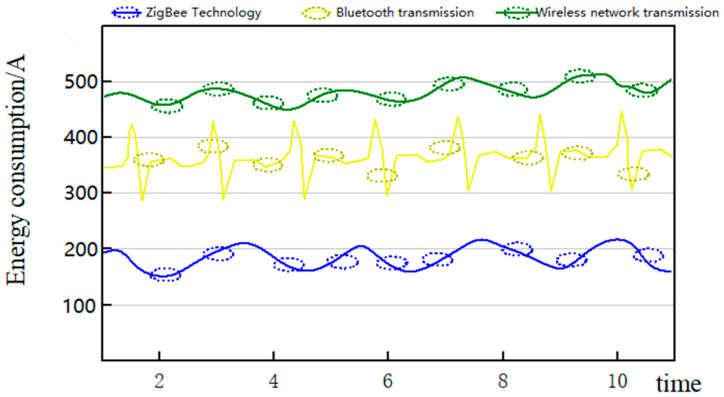
Three wireless networks’ data transmission energy consumption.

It can be seen from Table 1 that ZigBee technology is more flexible in terms of the scope of use of the network and can increase the coverage and communication distance at any time. We can find it under the same transmission distance. In terms of energy and power consumption, ZigBee technology can transmit more data information with less energy. To further reflect the advantages of ZigBee technology in terms of energy consumption, we compare the data transmission energy consumption of three wireless networks in the same time range, as shown in Figure 5.

**Figure 6:**
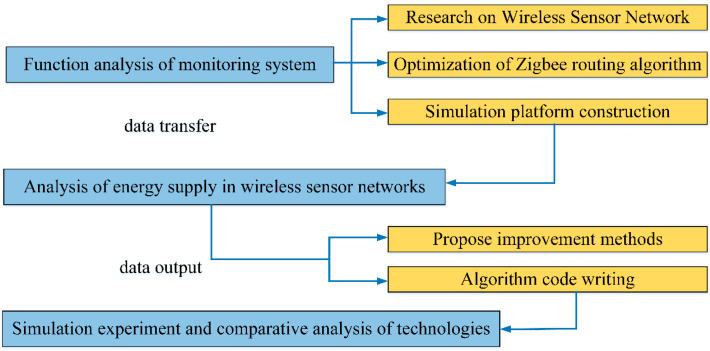
Mainline optimization process.

It can be seen from Figure 5 that ZigBee technology and the other two technologies consume significantly different amounts of system energy under the same data transmission volume. We can find these differences under the same transmission distances. As data are continuously transmitted, the energy consumption of transmission using ZigBee technology is the lowest of the three types of transmission and remains highly stable over time. ZigBee technology is more suitable for the purpose of low power consumption. If it is used in the monitoring system, the working time of the system can be extended. However, in order to meet the comprehensive coverage of the monitoring system, multiple sensor nodes need to be deployed. Each sensor needs to consume energy, and the total consumption has a heavy burden on the monitoring system. The data transmission of the network is also complex. If the network is blocked, the battery energy consumption will be accelerated. Therefore, we need to design an energy-optimized routing algorithm based on ZigBee technology to improve the energy supply effect of the system. The main optimization process of this study is shown in Figure 6.

It can be seen from Figure 6 that, firstly, the work structure of the monitoring system is analyzed, and the wireless sensor network module is used to process multiple tasks. From the energy defect of this technology, network routing and basic protocols are improved. According to the construction of the platform and protocol, energy optimization issues are proposed, and targeted suggestions are proposed for energy consumption, data transmission delay, etc. Finally, the algorithm’s simulation analysis and research examples’ design are completed, and a system platform is built to compare the performance of ZigBee energy optimization technology. When optimizing the energy and transmission, the topology of the ZigBee wireless sensor network should be analyzed first. We embody the particularity of the technology from the two modes of the tree network and star network.

**Figure 7:**
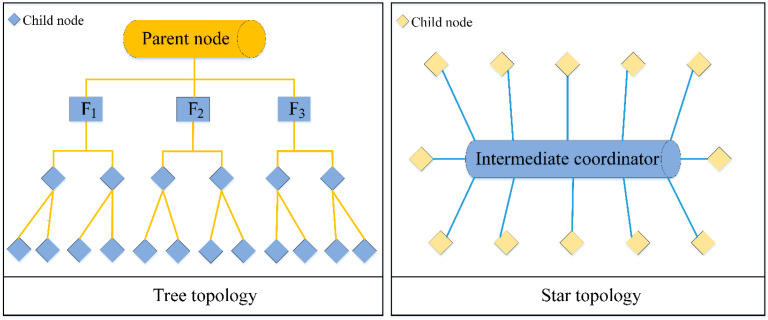
Tree network, star network structure.

It can be seen from Figure 7 that the tree network is a special structure in a ZigBee wireless network system. Each sensor node is based on the parent node and completes network deployment through the distribution of child nodes. However, this structure has high requirements for the central parent node, and the failure here is likely to cause the entire network to be paralyzed. The star network takes the intermediate coordinator as the core, connecting all sensor nodes with the coordinator. Each subnode cannot complete the data transmission task, and can only upload to the central controller for forwarding. Although this network structure is relatively simple, it also depends on the working state of the central coordinator. Therefore, we removed the above two types from the ZigBee network selection and choose a topology scheme that combines mesh structure with tree structure. During data and information transmission, resource sharing is realized through node interconnection, and the combination between points is realized according to the topology structure, which is more suitable for monitoring systems under wireless sensor networks from scalability to use cost [37]. After analyzing the network structure of ZigBee technology, we analyze its network address allocation in detail. Assuming that the maximum number of child nodes that can be connected in the network node is fixed, and the number of routes is *C_x_*, the address offset can be obtained according to the following formula:

(1) $$S\left(o\right)=C_{x}\left(L_{y}-d\right)+\left(1-R_{x}\right)/1-C_{u}$$

The formula represents the offset factor. After the subnodes of the same type join the network structure, we calculate that the address assigned to this node is:

(2) $$M=k_{x,y}+d\left(m\right)+l,0\leq l\leq d$$

If the joined node has its own route, we reallocate the address from the parent node to the child node:

(3) $$M=k_{x,y}\left[1+d\left(m\right)\right]/\left(o-u\right)$$

From the above formula, you can obtain a calculated instance path. Next, the data transmission of network nodes is scheduled according to the clustering tree routing algorithm. We upload the network data according to the relationship between the child node and the parent node in turn, and finally form a data transportation rule. This static routing only needs to ensure that the relationship between nodes is orderly, avoiding the problem of resource consumption when the route is updated. When judging whether a node has reached the parent node branch, it can be calculated by the following formula:

(4) $$D\left(C_{u}-K_{p}\right)=x,y,l$$

(5) $$D>R=u+l_{m}\left(k_{x-y}\right)$$

In the formula, *K_p_* represents the distance coefficient for data transmission. If the child node location is found to be incorrect, the formula for retransmitting data is:

(6) $$C=N_{0}+\left[F_{k}-\left(d_{0}+l\right)/m_{x,y}\right]\left(l_{d}\right)$$

This clustering tree routing is relatively simple and suitable for devices with low power consumption in wireless sensors. It can also make up for the lack of energy supply of the monitoring system to a certain extent. However, due to the low efficiency of routing, the established data transmission path is not the best solution, so there will be a large delay problem when facing a large number of dynamic data. Therefore, in the experimental design, we also introduced the path optimization scheme to improve the local network and realized the energy optimization in the ZigBee network. Check the node location information before and after data transmission, and reduce the network transportation cost and consumption according to the optimal path strategy. This improves the performance of wireless sensor networks, reduces the total consumption of the whole network during system operation, and extends the working time and service life. Next, we also studied the optimization of embedded cloud computing in data processing to further improve the performance and reliability of the monitoring system in processing dynamic data information.

### 3.2. Simulation of ZigBee-Optimized Wireless Sensor Network in Embedded Cloud Computing Data Monitoring System

**Figure 8:**
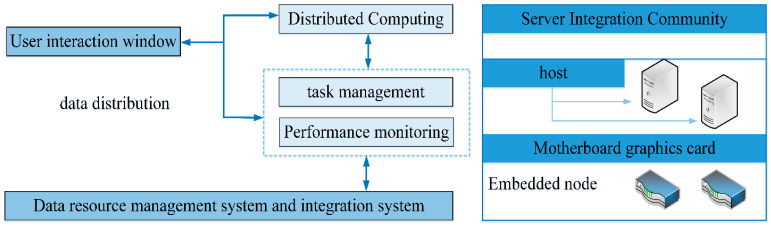
Computing mode internal architecture.

While the access side network and computing capabilities are improved, the capabilities of many terminals can be further optimized in the future. Some general businesses can be moved to the cloud computing data monitoring system platform. This will significantly simplify terminal implementation and reduce the difficulty of business implementation and operating costs. At that time, the 6G network will develop into an integrated network integrating the cloud, network, edge, end, and use on the access side. In many fields, the support of a monitoring system is needed to complete the task and purpose. It can be seen that video monitoring and data monitoring have become the primary means of urban security and construction protection. When the coverage of the monitoring system gradually increases, the demand for data collection and analysis also grows. The traditional monitoring system is small in scale and prone to low efficiency and error when processing dynamic data. Therefore, the data monitoring system under the embedded cloud computing mode provides practical help for target detection, target tracking, traffic monitoring, police monitoring, and other links. Data monitoring has started to develop in the direction of massive, intelligent, fast processing technology. The embedded cloud computing platform takes its virtualization and integration as the management basis of monitoring information. It can be effectively combined with wireless sensor networks to form the interconnection effect between multi-platform nodes. The internal architecture of this high-performance computing mode is shown in Figure 8.

**Figure 9:**
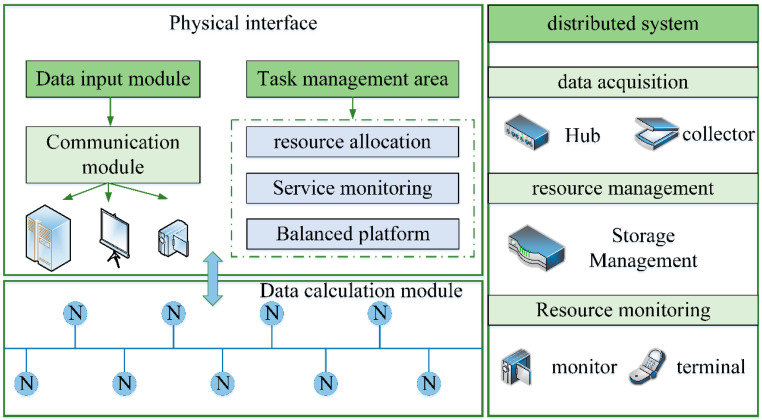
Real-time data processing framework and distributed system architecture.

It can be seen from Figure 8 that the internal architecture of the platform is composed of a user interaction window, data calculation, task management, system monitoring, resource management, and other modules. Among them, the server integration group also includes the label of the host and motherboard models, connecting the chip with the board according to the embedded system model. To enable the data monitoring system to cover a broader range, we also need to deploy nodes with the help of wireless sensor networks and set up connection points at each key location in public places. The energy optimization improvement of ZigBee technology has been carried out for the wireless sensor network above to reduce the energy consumption of the monitoring system in actual operation. This composite embedded cloud computing system is characterized by high real-time, low power consumption, simple deployment, and high connectivity with sensor nodes. At the same time, this system’s distributed real-time computing capability can process a large amount of information in the monitoring data. We show the real-time data processing framework and the distributed system structure, as shown in Figure 9.

It can be seen from Figure 9 that the real-time calculation module of the data monitoring system mainly consists of the physical interface, data link input, task management, resource allocation, and other functions. A wireless sensor communication network is interspersed between each module, which is responsible for the data reception and transmission between deployment nodes. To ensure the information quality in data monitoring, we define the concept of data quality according to different sensing methods:

(7) $$F=Q\cdot \left(R_{-}G\right)$$

(8) $$F=\left\{\left(T_{0},G_{1}\right),\left(T_{1},G_{2}\right),\ldots ,\left(T_{m-1},G_{m}\right)\right\}$$

Each element in the data set represents that the uploaded monitoring information is within a quality range and defines different data types, such as text, video, image, sound, etc. Among them, set definitions are performed for different data types:

(9) $$Z=f\left(Q\cdot R_{m}\right)$$

In the formula, *Z* represents the video monitoring data type. We find that in practical applications, most of the data uploaded by monitoring is video based. Therefore, in computing tasks, we can find the average value of the quality between videos:

(10) $$G=\left[d_{0},d_{1},\ldots ,d_{m}\left(R\cdot f\right)\right]$$

For each data node, the formula for the average processing time is:

(11) $$A_{v}=\left\{E_{ps,s},\ldots ,E_{ps,m}\right\}\cdot F$$

(12) $$s=D\cdot R\left[t_{0}+\left(Q_{s}-t\right)\right]/m+n$$

In the formula, *s* represents the total time spent processing surveillance video data. Based on the above definitions, the system running time required for long videos in each processing set is calculated:

(13) $$p=e_{sp,g}\cdot ts+W$$

(14) $$p_{1}=e_{ps,g}\left[ts\cdot \left(W+d\right)/R_{p1}\right]+ts_{0}\cdot w_{p}$$

Among them, *p*_1_ represents the time node for uploading data, and *R_p_*_1_ the time node represents the end of video data processing. Because of the combination of embedded cloud computing and wireless sensor networks, it is easy to process duplicate information when computing large amounts of data. Therefore, we clean up the data of multiple associated nodes before calculating the system runtime:

(15) $$T_{R}=\sqrt{M\cdot P_{i}/m+t_{0}s}$$

**Figure 10:**
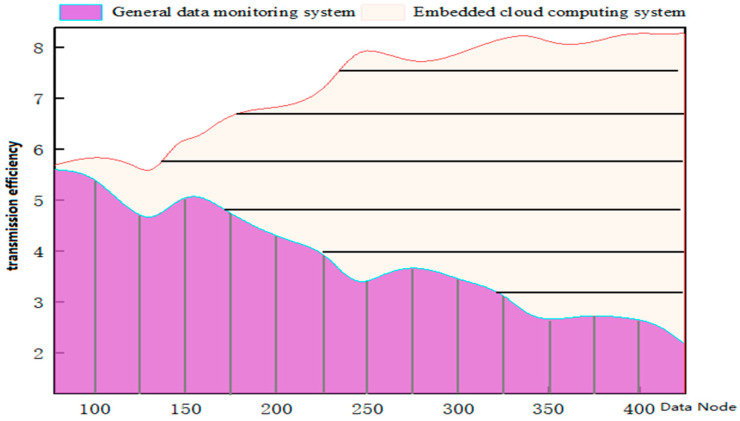
Transmission efficiency between ordinary data monitoring system and embedded cloud computing system.

According to the above formula, we ran the data monitoring system in practical applications and found that the running speed of embedded cloud computing was significantly improved. We compared the efficiency of ordinary data monitoring systems and the embedded cloud computing system in multi-task and multi-node data transmissions, as shown in Figure 10:

It can be seen from Figure 10 that the dynamic data uploaded by the wireless sensor network in the face of the ordinary data monitoring system has a fault phenomenon in the transmission process. The real-time data transmission system using embedded cloud computing distributed processing can effectively solve the dynamic information uploaded by different network nodes and improve the system transmission efficiency.

## 4. Research Results’ Analysis of Embedded Cloud Computing Data Monitoring System Based on ZigBee Energy Optimization Routing Algorithm for Wireless Sensor Networks

### 4.1. Analysis of Simulation Results of Wireless Sensor Network Routing Algorithm Based on ZigBee Energy Optimization

With powerful AI and extensive data analysis and calculation, the 6G network will become a computing and data network for the converged cloud, network, edge, and end. The hardware in the 6G network can rely on the unique hardware platform, general hardware platform, open source hardware platform, etc. We are adopting different degrees of open source and open strategies to decouple network elements and functions deeply. Resources will help build a universal and open network architecture for customized services, support the reconfiguration and plug-and-play of 6G networks, meet the needs of telecom operators for efficient and scalable networks, flexible and diversified services, and open industry ecology, and thus promote the business development of vertical applications. Since the cloud server cannot reach the bandwidth we need, we deployed the embedded cloud platform monitoring system and the simulation model on the physical server and installed a 10 GbE network card. The download and upload speed can reach about 10 Gbps simultaneously due to the short transmission distance and very low latency. In the experiment, the speed and delay standards of the 6G network environment can be achieved. ZigBee technology is a short-range wireless communication technology with low power consumption. The industry considers it most likely to be used in industrial control applications of wireless methods. It also uses the 2.4 GHz band, frequency hopping technology, and spread spectrum technology. The most important thing is that it can be networked with 254 nodes. The characteristics of ZigBee technology allow it to have great potential for industrial monitoring, sensor networks, home monitoring, security systems, and other fields. In security performance, ZigBee adopts the encryption algorithm of AES-128, and each application can flexibly determine its security attributes. It provides a data packet integrity check function based on cyclic redundancy check (CRC) and supports authentication. Although the traditional wireless sensor network can achieve fast data transmission in a small range, it has apparent defects in the node data transmission and power consumption stability. We compared various methods from wireless communication and sensor technology and chose ZigBee technology as the energy optimization method of the wireless sensor network. ZigBee energy optimization adopts a clustering routing algorithm to find the best sensor node path through the routing target before data transmission to reduce the energy loss caused by invalid paths during system operation. However, ordinary routing optimization needs to add all network nodes to the calculation, and most packets have no practical significance. This large-scale inaccurate calculation results in many redundant data, which increases energy consumption. Thus, we added the AODVjr algorithm as the fusion part to find the best solution for sensor nodes. In the AODVjr algorithm, many data packet controls exist, including route response, route request, and route error return. We show the process of this algorithm in monitoring data transmission.

**Figure 11:**
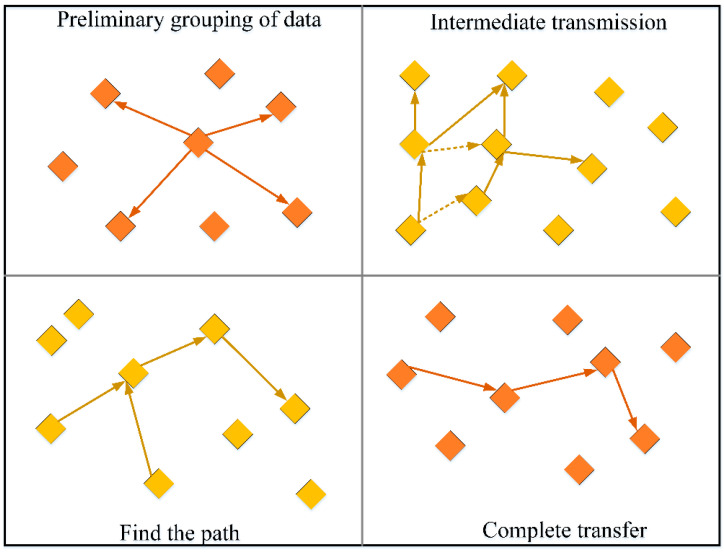
This algorithm monitors the process of data transmission.

As shown in Figure 11, the schematic diagram shows four modes of primary data grouping, intermediate transmission, pathfinding, and transmission completion. After receiving the packet data, each sensor node first judges whether there is the same type of data information as the root destination node, and the transit node sends the data in packets. If there are traces of history, it is optional whether to re-establish the path or to complete the transmission according to the historical path. If the sensor node does not find a suitable route and path, it immediately sends a broadcast request to other parts to find the destination. Therefore, the routing transmission path selected by this algorithm is the minimum consumption path for system operation and has a good effect on the application of large-scale wireless sensor networks. We compared the performance of the three transmission modes of the clustering routing algorithm, AODVjr algorithm, and fusion algorithm in terms of energy consumption, response time, transmission efficiency, etc., as shown in Table 2.

**Figure 12:**
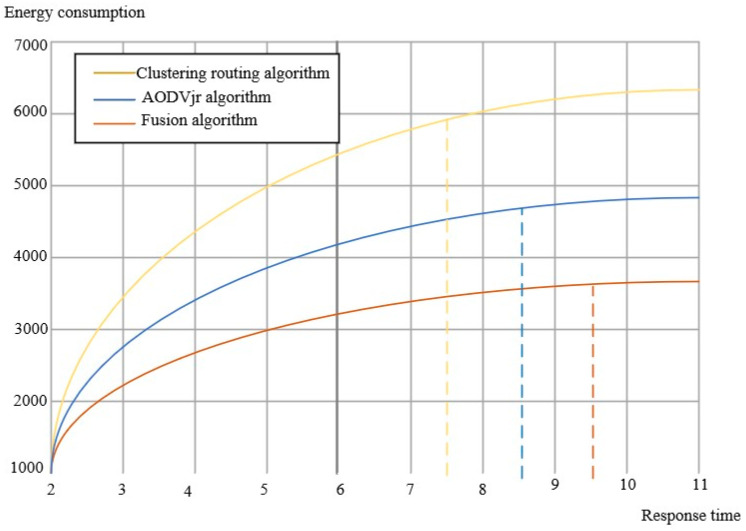
Energy consumption change of three algorithms.

Table 2 shows that the energy consumption of the clustering routing algorithm and AODVjr algorithm is medium, while the energy consumption of the fusion algorithm is the minimum. At the same time, the three algorithms are further analyzed in terms of energy consumption, as shown in Figure 12.

**Figure 13:**
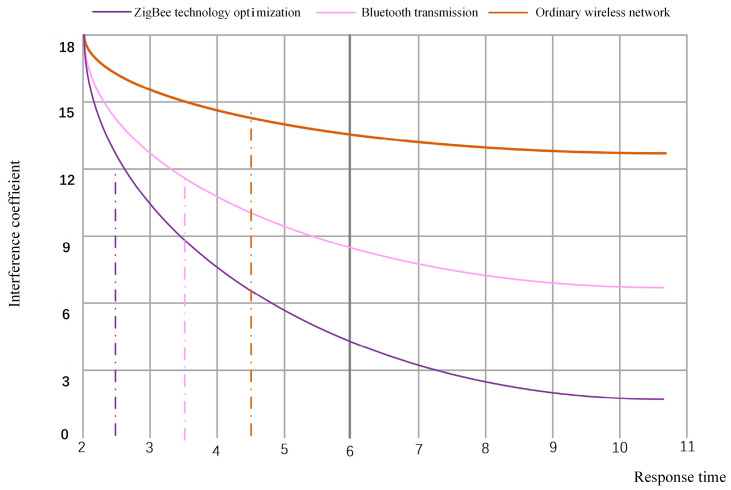
Data anti-interference changes of three networks.

It can be further proved from Figure 12 that ZigBee energy optimization can reduce the energy consumption of the sensor network during operation under the fusion of the two routing algorithms. We can see that the energy consumption gradually stabilizes after 9 S of running the simulation model. The fused algorithm consumes about 1000 A less than the AODVjr algorithm alone and about 2000 A less than the clustering routing algorithm alone. In terms of response and system feedback duration and transmission efficiency, the algorithm is significantly better after integrating the two. Due to the high cost of verifying ZigBee technology, we used simulation to prove it. NS2, open source-oriented software, was selected in the simulation platform construction and combined with C language high-level execution compilation to build the model mechanism jointly. Finally, we compared the data anti-interference of the ZigBee energy-optimized sensor network with Bluetooth transmission and ordinary wireless network transmission on the simulation platform, as shown in Figure 13.

It can be seen from Figure 13 that there are apparent interference effects when the response time of ordinary wireless networks and Bluetooth transmission reaches 2 S. The wireless sensor network optimized by ZigBee technology has a longer anti-interference time and more obvious advantages than the other two. This research not only makes a comparison of multiple routes in terms of energy consumption but also shows a clear distinction in terms of data transmission efficiency and transmission accuracy. Therefore, it proves that a ZigBee energy-optimized wireless sensor network can be effectively applied in the data monitoring system.

### 4.2. Analysis of Simulation Results of ZigBee-Optimized Wireless Sensor Network in Embedded Cloud Computing Data Monitoring System

**Figure 14:**
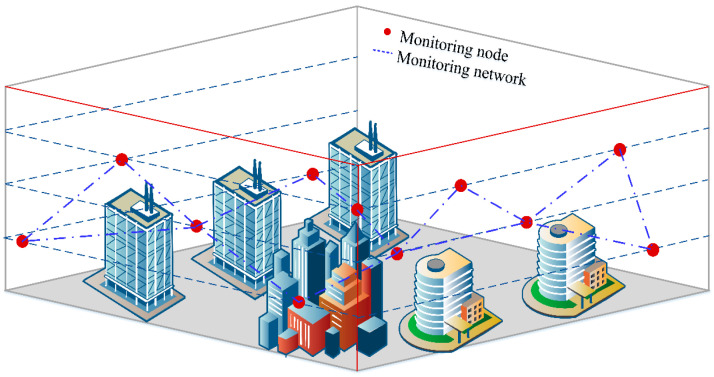
Virtual model of monitoring scope.

To support the application of intelligent services, the network layer needs to perceive the services of the application layer to complete the intelligent and automatic management, scheduling, and allocation of computing resources in all parts of the network. In the 6G era, the network may realize the unified management of physical machines, virtual machines, containers, and other cloud network infrastructure resources by building a unified cloud network integration operating system; by building a unified network and application orchestration system to achieve on-demand orchestration and invocation of network functions and business applications; and by combine edge computing and edge AI capabilities to achieve intelligent data analysis and governance. Cloud computing and embedded data processing are combined through multiple tasks, parallel processing, distributed structure, and other combinations, which can provide users with more convenient, fast, and intelligent services. In cloud monitoring and large-scale video monitoring tasks, the embedded cloud system platform can directly connect the monitoring sensor node with the management end. This real-time interactive system is more in line with the needs of modern society. After the server is connected to the cloud system, it can handle a variety of data types uploaded by the physical monitoring layer to achieve the purpose of resource sharing and information analysis. The traditional monitoring system can only complete the monitoring task through a single sensor node when it is initially deployed. The data monitoring platform is developing towards a wider range with the introduction of wireless sensor networks. We built a virtual model of the data monitoring range under the wireless sensor network, as shown in Figure 14.

**Figure 15:**
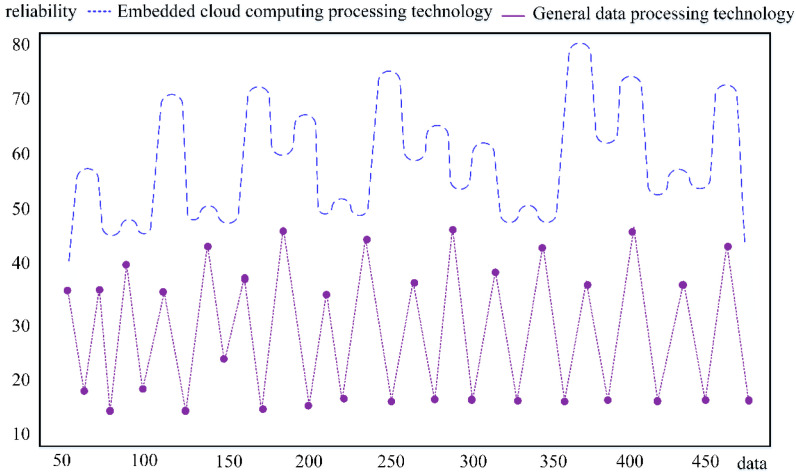
Comparison of data transmission reliability between the two.

It can be seen from Figure 14 that the monitoring layout nodes of a certain urban area are randomly selected, and the network system formed by the monitoring nodes is displayed using virtual simulation software and a satellite positioning system. We find that in modern urban construction, the distribution of monitoring systems is becoming larger and larger, and each node is connected with the other. In this dynamic data environment, embedded cloud computing processing can carry a large amount of computing workload. In providing data analysis services for monitoring users, embedded cloud computing technology also has strong reliability. It can not only protect the effectiveness of data transmission but also maintain system security and reduce the probability of external intrusion. At the same time, the restrictions of time and space are broken to realize the sensing connection within the region and city. This data monitoring system can complete data transmission across regions and platforms and has obvious advantages in target searching, character screening, target tracking, and other functions. We used the virtual simulation network to compare the embedded cloud computing processing with the ordinary data processing technology, and analyzed the data transmission reliability of the two in the process of monitoring the change in information volume, as shown in Figure 15.

**Figure 16:**
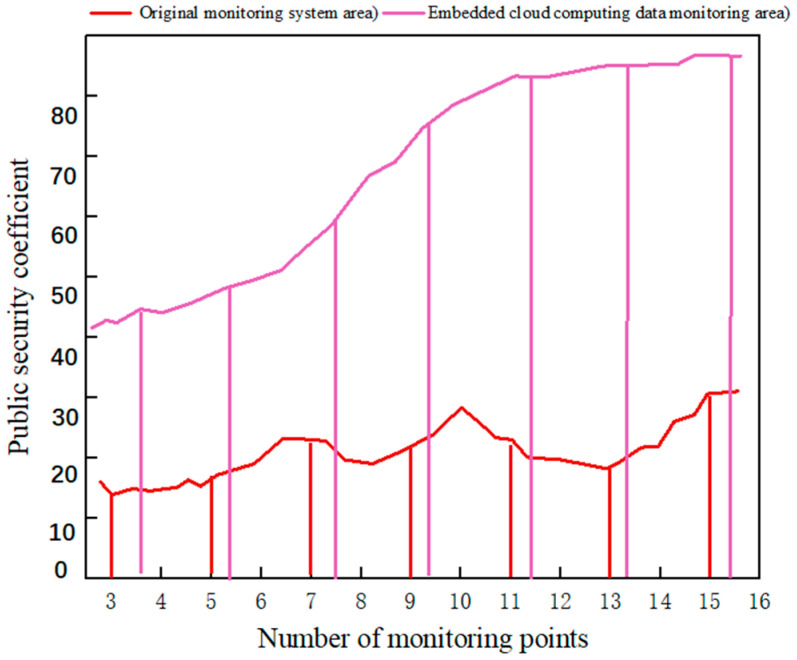
Comparison of urban public security coefficients between two monitoring systems.

It can be seen from Figure 15 that as the amount of data uploaded in the network environment increases, more and more complex information is integrated into it, which hinders the standard data processing system. The reliability of data processed by embedded cloud computing is significantly higher than that of ordinary data processing. This data transmission efficiency is also reflected in the distributed design within the system, which allocates the data uploaded by multiple nodes in the wireless sensor network to tasks. The complex task is divided into several small computing modules. Finally, data processing results are extracted, summarized, and uploaded to the monitoring terminal as information support. Since ZigBee energy optimization is used in wireless sensor network access, the data monitoring system can support a broader range of data transmission and work. Finally, using the simulation model, we applied the embedded cloud computing data monitoring system under the wireless sensor network to the urban area. We compared the impact of the original monitoring system and the optimized data monitoring system on the city security coefficient, as shown in Figure 16.

It can be seen from Figure 16 that the urban safety factors using the original monitoring system and the optimized data monitoring system changed significantly. With the continuous increase in monitoring points, the safety factor of the city also gradually improved, and the system performance does not cause energy shortages and network problems due to the increase in monitoring nodes. The data monitoring system using embedded cloud computing and a wireless sensor network can improve the urban security environment and ensure people’s safe daily travel. At the same time, it provides practical help for traffic management, police assistance, target tracking, and other work.

The development of 6G will realize the coordinated development of terminals and networks and use wireless sensor networks to meet the needs of many intelligent terminals and application services for computing, storage, and services. The ubiquitous terminal requires high intelligence, strong computing power, and microservice capabilities. End–cloud collaboration can transfer this demand to the edge computing platform to reduce the manufacturing and holding costs of 6G ubiquitous intelligent terminals, thus effectively improving the popularity of 6G applications and services. Application scenarios should guide the development of end–cloud collaboration. Based on the background of the deep integration and close collaboration of the cloud and network edge, the industry should not only accelerate the construction of end–cloud collaboration standard systems, but we also need to start from the overall layout of 6G, based on the network, and conduct research and design under the framework of the central cloud, edge cloud, and 6G ubiquitous intelligent terminals.

## 5. Conclusions

The construction of the 6G network will be based on the microservice architecture. For the basic service unit, the network needs to meet the differentiated needs of vertical industries and combine network capacity opening, cloud edge collaboration, and intelligent analysis to achieve different levels of capacity opening and network industry integration. As the monitoring system develops toward the goal of large-scale, big data and intelligence, many technologies for processing system data will also need to begin to progress towards the direction of science and technology. The traditional monitoring mode can only complete a single data transmission, which cannot meet the needs of modern cities in terms of layout scope, working hours, data transmission efficiency, data security, etc. Therefore, wireless sensor networks and embedded cloud computing systems become the primary means to solve the above problems. Cloud computing and embedded technology have noticeable effects in monitoring data and reconstructing video information. This distributed system processing method also provides help for extensive data analysis. Standard wireless sensor networks have apparent defects in node distribution and work continuity. This paper proposes improving the energy supply and service life of wireless sensor networks based on ZigBee energy optimization technology and improving the data monitoring systems’ distribution range. First, the ZigBee technology is analyzed, and a clustering algorithm is proposed to optimize the routing protocol for high energy consumption. In the optimization process, the AODVjr algorithm is added as the comparison result of energy consumption. It is found that the two optimization methods cannot significantly reduce the unnecessary consumption generated by the operation of the monitoring system. Therefore, the two algorithms are integrated into ZigBee technology to reduce data redundancy and repeated processing times during the use of wireless sensor networks and reduce network consumption. Finally, embedded cloud computing is used to distribute the computing data content with multiple data types as the boundary when processing the monitoring data. The dynamic monitoring data are added to the process of multi-task joint processing, which improves the cloud storage of the monitoring system and reduces the response time of processing monitoring data. The results show that using ZigBee energy to optimize wireless sensor networks in embedded cloud computing data monitoring systems can improve system operation efficiency. It not only reduces the running loss of the system but also ensures the security of the data and realizes a wide range of monitoring coverage. As ZigBee energy optimization is used for wireless sensor network access, the data monitoring system can support a broader range of data transmission and work. Finally, we use the simulation model to apply the embedded cloud computing data monitoring system under wireless sensor networks to urban areas. The analysis shows that the data monitoring system using embedded cloud computing and a wireless sensor network can improve the urban security environment. Although the experimental results of this study are relatively good in the simulated simulation environment, it is currently impossible to detect and verify the energy consumption in the actual data transmission due to the limitations of the physical environment, which is our next research goal.

Due to the current global development of 6G, first of all, we need to meet the more extensive bandwidth, lower latency, and higher rate to realize the interconnection of hundreds of millions of various devices to meet the geometrically dramatic increase in data traffic, and our spectrum resources are becoming increasingly tight. Moreover, due to the lack of maturity of the chip and physical layer technology, we cannot reach the terahertz band, the visible light band, etc. On the other hand, the lack of energy efficiency of the current communication network will cause a variety of types of energy conversion waste, including photoelectric conversion, resulting in a large amount of energy loss for long-distance transmission within cities or information transmission between cities. However, with the current progress of various network communication and chip technologies, the continuous evolution of 6G, and the integration of new services, a new era of communication can be realized.

## Figures and Tables

**Table 1:** Transmission Performance Of Three Technologies.

Type of Technology	Transmission Distance/M	Transmission Efficiency/%	Security/%	Energy Consumption
Zigbee Technology	150	90	98	300
Bluetooth Transmission	150	78	60	465
Wireless Network Transmission	150	80	58	372

**Table 2:** Performance Comparison Table of Three Algorithms.

Algorithm Type	Energy Consumption/A	Response Time/S	Transmission Efficiency
Clustering Routing Algorithm 1	5300	10	500
Clustering Routing Algorithm 2	5800	11	514
AODVjr Algorithm 1	4520	8	654
AODVjr Algorithm 2	4652	9	687
Fusion Algorithm	2003	3	1540

## References

[1] Tolani M., Singh R.K., Shubham K., Kumar R. (2017). Two-layer optimized railway monitoring system using Wi-Fi and ZigBee interfaced wireless sensor network. IEEE Sens. J..

[2] Jung H.J., Song Y., Hong S.K., Yang C.H., Hwang S.J., Jeong S.Y., Sung T.H. (2015). Design and optimization of piezoelectric impact-based micro wind energy harvester for wireless sensor network. Sens. Actuators A Phys..

[3] Ouni S., Ayoub Z.T. (2013). Cooperative association/re-association approaches to optimize energy consumption for real-time IEEE 802.15. 4/ZigBee wireless sensor networks. Wirel. Pers. Commun..

[4] Peng C., Qian K., Wang C. (2014). Design and application of a VOC-monitoring system based on a ZigBee wireless sensor network. IEEE Sens. J..

[5] Jawad H.M., Jawad A.M., Nordin R., Gharghan S.K., Abdullah N.F., Ismail M., Abu-AlShaeer M.J. (2019). Accurate empirical path-loss model based on particle swarm optimization for wireless sensor networks in smart agriculture. IEEE Sens. J..

[6] Wadhwa L.K., Deshpande R.S., Priye V. (2016). Extended shortcut tree routing for ZigBee based wireless sensor network. Ad Hoc Netw..

[7] Govindasamy J., Punniakody S. (2018). A comparative study of reactive, proactive and hybrid routing protocol in wireless sensor network under wormhole attack. J. Electr. Syst. Inf. Technol..

[8] Sundhari RP M., Jaikumar K. (2020). IoT assisted Hierarchical Computation Strategic Making (HCSM) and Dynamic Stochastic Optimization Technique (DSOT) for energy optimization in wireless sensor networks for smart city monitoring. Comput. Commun..

[9] Yu Y., Xue B., Chen Z., Qian Z. (2019). Cluster tree topology construction method based on PSO algorithm to prolong the lifetime of ZigBee wireless sensor networks. EURASIP J. Wirel. Commun. Netw..

[10] Mahmood A., Khan I., Razzaq S., Najam Z., Khan N.A., Rehman M.A., Javaid N. (2014). Home appliances coordination scheme for energy management (HACS4EM) using wireless sensor networks in smart grids. Procedia Comput. Sci..

[11] Tahmassebpour M. (2016). Methods and algorithms of capacity calculation and increase throughput in wireless sensor networks base of ZigBee: A survey. Indian J. Sci. Technol..

[12] Rault T., Bouabdallah A., Challal Y. (2014). Energy efficiency in wireless sensor networks: A top-down survey. Comput. Netw..

[13] Bhushan S., Kumar M., Kumar P., Stephan T., Shankar A., Liu P. (2021). FAJIT: A fuzzy-based data aggregation technique for energy efficiency in wireless sensor network. Complex Intell. Syst..

[14] Bianchi V., Ciampolini P., De Munari I. (2018). RSSI-based indoor localization and identification for ZigBee wireless sensor networks in smart homes. IEEE Trans. Instrum. Meas..

[15] Abella C.S., Bonina S., Cucuccio A., D’Angelo S., Giustolisi G., Grasso A.D., Scuderi A. (2019). Autonomous energy-efficient wireless sensor network platform for home/office automation. IEEE Sens. J..

[16] Wan B.F., Zhou Z.W., Xu Y., Zhang H.F. (2020). A theoretical proposal for a refractive index and angle sensor based on one-dimensional photonic crystals. IEEE Sens. J..

[17] Zhao L., Qu S., Yi Y. (2018). A modified cluster-head selection algorithm in wireless sensor networks based on LEACH. EURASIP J. Wirel. Commun. Netw..

[18] Mazinani A., Mazinani S.M., Mirzaie M. (2019). FMCR-CT: An energy-efficient fuzzy multi cluster-based routing with a constant threshold in wireless sensor network. Alex. Eng. J..

[19] Singh R.R., Yash S.M., Shubham S.C., Indragandhi V., Vijayakumar V., Saravanan P., Subramaniyaswamy V. (2020). IoT embedded cloud-based intelligent power quality monitoring system for industrial drive application. Future Gener. Comput. Syst..

[20] Chowdhury S.M., Hossain A. (2020). Different energy saving schemes in wireless sensor networks: A survey. Wirel. Pers. Commun..

[21] Govindarajan R., Meikandasivam S., Vijayakumar D. (2019). Cloud computing based smart energy monitoring system. Int. J. Sci. Technol. Res..

[22] Pereira R.I., Dupont I.M., Carvalho P.C., Jucá S. (2018). IoT embedded linux system based on Raspberry Pi applied to real-time cloud monitoring of a decentralized photovoltaic plant. Measurement.

[23] Atayero A.A., Williams R., Badejo J.A., Popoola S.I. (2019). Cloud based IoT-enabled solid waste monitoring system for smart and connected communities. Int. J. Civ. Eng. Technol..

[24] Nilsaz D.N., Barati H. (2020). A distributed energy-efficient approach for hole repair in wireless sensor networks. Wirel. Netw..

[25] Karimi B.S., Guo J., Jafarkhani H. (2020). Energy-efficient node deployment in heterogeneous two-tier wireless sensor networks with limited communication range. IEEE Trans. Wirel. Commun..

[26] Guleria K., Verma A.K. (2019). Comprehensive review for energy efficient hierarchical routing protocols on wireless sensor networks. Wirel. Netw..

[27] Mohamed R.E., Saleh A.I., Abdelrazzak M., Samra A.S. (2018). Survey on wireless sensor network applications and energy efficient routing protocols. Wirel. Pers. Commun..

[28] Li S., Kim J.G., Han D.H., Lee K.S. (2019). A survey of energy-efficient communication protocols with QoS guarantees in wireless multimedia sensor networks. Sensors.

[29] Almazaideh M., Levendovszky J. (2020). Novel reliable and energy-efficient routing protocols for wireless sensor networks. J. Sens. Actuator Netw..

[30] Jafarali Jassbi S., Moridi E. (2019). Fault tolerance and energy efficient clustering algorithm in wireless sensor networks: FTEC. Wirel. Pers. Commun..

[31] Selvi M., Santhosh Kumar SV N., Ganapathy S., Ayyanar A., Khanna Nehemiah H., Kannan A. (2021). An energy efficient clustered gravitational and fuzzy based routing algorithm in WSNs. Wirel. Pers. Commun..

[32] Harizan S., Kuila P. (2019). Coverage and connectivity aware energy efficient scheduling in target based wireless sensor networks: An improved genetic algorithm based approach. Wirel. Netw..

[33] Alsharif M.H., Kim S., Kuruoğlu N. (2019). Energy harvesting techniques for wireless sensor networks/radio-frequency identification: A review. Symmetry.

[34] Gudla S., Kuda N.R. (2022). Learning automata based energy efficient and reliable data delivery routing mechanism in wireless sensor networks. J. King Saud Univ.-Comput. Inf. Sci..

[35] Saba T., Haseeb K., Ud Din I., Almogren A., Altameem A., Fati S.M. (2020). EGCIR: Energy-aware graph clustering and intelligent routing using supervised system in wireless sensor networks. Energies.

[36] Song H., Sui S., Han Q., Zhang H., Yang Z. (2020). Autoregressive integrated moving average model–based secure data aggregation for wireless sensor networks. Int. J. Distrib. Sens. Netw..

[37] Kanoun O., Bradai S., Khriji S., Bouattour G., El Houssaini D., Ben Ammar M., Viehweger C. (2021). Energy-aware system design for autonomous wireless sensor nodes: A comprehensive review. Sensors.

